# Automated determination of bone age and bone mineral density in patients with juvenile idiopathic arthritis: a feasibility study

**DOI:** 10.1186/s13075-014-0424-1

**Published:** 2014-08-27

**Authors:** Janneke Anink, Charlotte M Nusman, Lisette WA van Suijlekom-Smit, Rick R van Rijn, Mario Maas, Marion AJ van Rossum

**Affiliations:** Department of Pediatrics/Pediatric Rheumatology, Sophia Children’s Hospital, ErasmusMC, Wytemaweg 80, 3015 CN Rotterdam, The Netherlands; Department of Radiology, Academic Medical Center, Meibergdreef 9, 1105 AZ Amsterdam, The Netherlands; Department of Pediatric Hematology, Immunology, Rheumatology and Infectious Disease, Emma Children’s Hospital, Academic Medical Center, Meibergdreef 9, 1105 AZ Amsterdam, The Netherlands; Department of Pediatric Rheumatology, Reade location Jan van Breemen, Dr. Jan van Breeemenstraat 2, 1056 AB Amsterdam, The Netherlands

## Abstract

**Introduction:**

Chronic inflammation combined with glucocorticoid treatment and immobilization puts juvenile idiopathic arthritis (JIA) patients at risk of impaired growth and reduced bone mineral density (BMD). Conventional methods for evaluating bone age and BMD are time-consuming or come with additional costs and radiation exposure. In addition, an automated measurement of bone age and BMD is likely to be more consistent than visual evaluation. In this study, we aimed to evaluate the feasibility of an automated method for determination of bone age and (cortical) bone mineral density (cBMD) in severely affected JIA patients. A secondary objective was to describe bone age and cBMD in this specific JIA population eligible for biologic treatment.

**Methods:**

In total, 69 patients with standard hand radiographs at the start of etanercept treatment and of calendar age within the reliability ranges (2.5 to 17 years for boys and 2 to 15 years for girls) were extracted from the Dutch Arthritis and Biologicals in Children register. Radiographs were analyzed using the BoneXpert method, thus automatically determining bone age and cBMD expressed as bone health index (BHI). Agreement between measurements of the left- and right-hand radiographs and a repeated measurement of the left hand were assessed with the intraclass correlation coefficient (ICC). Regression analysis was used to identify variables associated with Z-scores of bone age and BHI.

**Results:**

The BoneXpert method was reliable in the evaluation of radiographs of 67 patients (radiographs of 2 patients were rejected because of poor image quality). Agreement between left- and right-hand radiographs (ICC = 0.838 to 0.996) and repeated measurements (ICC = 0.999 to 1.000) was good. Mean Z-scores of bone age (−0.36, *P* = 0.051) and BHI (−0.85, *P* < 0.001) were lower compared to the healthy population. Glucocorticoid use was associated with delayed bone age (0.79 standard deviation (SD), *P* = 0.028), and male gender was associated with a lower Z-score of BHI (0.65 SD, *P* = 0.021).

**Conclusions:**

BoneXpert is an easy-to-use method for assessing bone age and cBMD in patients with JIA, provided that radiographs are of reasonable quality and patients’ bone age lies within the age ranges of the program. The population investigated had delayed bone maturation and lower cBMD than healthy children.

**Electronic supplementary material:**

The online version of this article (doi:10.1186/s13075-014-0424-1) contains supplementary material, which is available to authorized users.

## Introduction

Chronic inflammatory diseases such as juvenile idiopathic arthritis (JIA) can influence bone development. Continuous exposure to inflammatory cytokines, together with glucocorticoid therapy, affects bone formation. This combined with decreased physical activity and pubertal delay puts JIA patients at risk of impaired growth and reduced bone mineral density (BMD) [1,2]. The pediatric rheumatologist needs to be aware of both the bone age and the BMD of JIA patients in order to take this into account in choosing the best therapy.

The assessment of bone age is usually made using the Greulich and Pyle atlas [3]. Dual-energy X-ray densitometry (DXA) is the most commonly used method of assessing BMD [4]. Recently, BoneXpert was developed, bringing back the use of radiogrammetry, one of the oldest methods for assessing BMD [5]. This new digital X-ray radiogrammetry (DXR) method combines the assessment of bone age with a radiogrammetric measurement of (cortical) BMD (cBMD) of the second to fourth metacarpal joints. The cBMD is expressed by BoneXpert as a Bone Health Index (BHI), in which cBMD is corrected for size. The BoneXpert method makes use of conventional radiographs of the hand, thereby making it attractive for pediatric use because of its simple application, relatively low costs [6]) and lower effective radiation dose compared with other methods. Normative data are available because the method was validated in a healthy pediatric population [7,8]. The application has not been tested in JIA patients, in whom long-standing inflammation of the wrist can lead to growth abnormalities and destruction of the bone, thereby possibly complicating the assessment of bone age and bone health. Utilization of the Dutch Arthritis and Biologicals in Children (ABC) register provides a unique way to evaluate this method in a severely affected JIA population eligible for biologic treatment.

Our objective in this study was to evaluate the feasibility of an automated method for the determination of bone age and cBMD in severely affected JIA patients. A secondary objective was to describe bone age and cBMD in this specific JIA population eligible for biologic therapy.

## Methods

### Patient selection

Patients from two centers participating in the Dutch ABC register who were prospectively enrolled between 1999 and 2012 were eligible for this study. Biologic-naïve patients were selected, starting etanercept, of whom a standard radiograph of both hands and wrist in the posteroanterior view was made at the start of treatment (that is, 1 year before to 3 months after starting etanercept). The participants’ calendar age had to be within the reliability range of the BoneXpert method (that is, 2.5 to 17 years for boys and 2 to 15 years for girls). Ultimately, the radiographs of 69 patients were eligible for automatic analysis by BoneXpert. A flowchart of the patient selection is provided in Additional file 1.

### Clinical data collection

The ABC register, a multicenter, prospective, observational study, aimed to include all children diagnosed with JIA in whom biologic treatment was being initiated. Informed consent was obtained from all patients older than 12 years of age, in addition to the parents or guardians of all patients younger than 16 years of age. The study protocol was centrally approved by the Medical Ethics Committee of the Erasmus MC, and local permission was obtained from the ethical bodies in the two other participating hospitals (Academic Medical Center and Reade). From the ABC register, patient and disease characteristics were collected at baseline, including data on disease activity from the following sources: physician’s global assessment of disease activity on a visual analogue scale (VAS) (range from 0 to 10 cm, with 0 being the best score); Childhood Health Assessment Questionnaire (CHAQ) (range from 0 to 3, with 0 being the best score) by patients and/or their parents, including global assessment of well-being and pain on a VAS (range from 0 to 10 cm, with 0 being the best score); number of joints with active arthritis and joints with limited motion; and erythrocyte sedimentation rate (ESR). Using these variables, the Juvenile Arthritis Disease Activity Score in 10 active joints (JADAS-10) was calculated. The scale from 0 to 40 represents the simple linear sum of the scores of the physician and parent and/or patient global assessment, an active joint count (up to 10 joints) and a normalized value of ESR to a 0 to 10 scale [9].

### Image analysis

The stand-alone Windows product of BoneXpert (BoneXpert Version 2.1.0.12; Visiana, Holte, Denmark) was used to analyze the hand radiographs. BoneXpert automatically generates the following outcome variables: (calendar) age, bone age based on Greulich and Pyle, Z-scores of bone age (compared with a healthy reference population) [10], BHI and Z-scores of BHI [5]. BHI is based on the cortical thickness (T) of the three middle metacarpals. In the construction of BHI, metacarpal width (W) and length (L) are also incorporated to compensate for the high variation in stature of growing children, as expressed in the following formula [5]:

$$ \mathrm{B}\mathrm{H}\mathrm{I} = \uppi \mathrm{T}\left(1\ \hbox{--}\ \mathrm{T}/\mathrm{W}\right)/{\left(\mathrm{L}\mathrm{W}\right)}^{0.33} $$

The radiographs included the complete hand and wrist joints of both the left and right sides. All images were collected as a DICOM files from three different centers. If the radiographs were available only on conventional films, they were digitized with a Sierra Plus scanner (VIDAR Systems, Herndon, VA, USA) and converted to a 300-dpi DICOM file. During the analytical process in BoneXpert, possible error messages were noted. The left-hand radiograph was uploaded and analyzed in BoneXpert a second time in order to be able to determine its test–retest reliability.

### Statistical analysis

Descriptive statistics are reported in terms of absolute numbers, median and interquartile range (IQR) or mean and standard deviation (SD). The single measure intraclass correlation coefficient (ICC) and Bland-Altman plots were used to determine the agreement of the outcome variables of the BoneXpert method.

To determine whether the Z-score of bone age and the Z-score of BHI were different from those in the healthy population, a one sample *t*-test was used. Univariate linear regression analysis was performed to identify variables associated with the Z-score of bone age and the Z-score of BHI. Because of the small sample size, only a limited number of variables could be tested. The prespecified variables entered into the univariate model were age, sex, JADAS-10, disease duration and use of systemic glucocorticoids, defined as “ever use” or “never use.” All analyses were performed with SPSS version 20 software (SPSS, Chicago, IL, USA).

## Results

### Feasibility

A standard hand radiograph of both hands was available for 69 patients starting etanercept treatment. The calculations of bone age and BMD by BoneXpert took a few seconds. BoneXpert rejected the radiographs of two patients because of poor image quality, resulting in available BoneXpert outcomes of 67 patients. In three patients, an error message indicating uncertainty of bone age was given by BoneXpert; these patients had calendar ages within the BoneXpert age ranges (2.5 to 17 years for boys and 2 to 15 years for girls), but their bone age came out of the analysis to be outside these age ranges, resulting in an error message. However, BoneXpert produced all outcome variables in these three patients, except for a missing Z-score of BHI in one patient.

The ICC of the agreement of all outcome variables between the left and right hand radiographs varied from 0.838 to 0.996. Bland-Altman plots of the agreement between left- and right-hand radiographs are provided in Additional file 2. The ICC of the agreement of repeated measurements of all left-hand radiographs on Z-scores of bone age and BHI varied from 0.999 to 1.000.

### Patient and disease characteristics

Patient and disease characteristics of the 67 patients who could be evaluated with BoneXpert are presented in Table 1. Disease activity of these patients was moderate to severe at the time the hand radiographs were made (mean JADAS-10 score = 21 ± 5).

**Table 1:** **Patient and disease characteristics at start of etanercept therapy** ^**a**^

**Baseline characteristics**	***N*** **= 67 patients**
Females, *n* (%)	36 (54)
Age at onset JIA in years, mean (±SD)	8.5 (±3.8)
Age at start etanercept in years, mean (±SD)	11.0 (±3.1)
JIA disease duration before start etanercept in years, median (IQR)	1.8 (1.1 to 3.8)
ANA-positive, *n* (%)	14 (21)
Category JIA, *n* (%)	
Systemic JIA	4 (6)
Polyarticular RF-negative	27 (40)
Polyarticular RF-positive	9 (13)
Oligoarticular extended	18 (27)
Psoriatic arthritis	5 (8)
Enthesitis-related arthritis	4 (6)
Previously used medications, *n* (%)	
Systemic prednisone	32 (48)
Methotrexate	66 (99)
DMARD other than MTX	14 (21)
Concomitant comedication at start of biologic therapy, *n* (%)	
Systemic prednisone	26 (39)
Methotrexate	64 (96)
DMARD other than MTX	2 (3)
Disease activity parameters at baseline, median (IQR)	
Physician-rated VAS (0 to 10 cm; best score = 0)	6.5 (5.0 to 7.4)
CHAQ total (0 to 3; best score = 0)	1.50 (0.90 to 2.10)
VAS pain (0 to 10 cm; best score = 0)	6.3 (2.4 to 7.7)
VAS well-being (0 to 10 cm; best score = 0)	6.1 (3.2 to 7.4)
Active joints	11 (7 to 18)
Limited joints	6 (3 to 13)
ESR	11 (5 to 29)
JADAS-10 (0 to 40), mean (±SD)	21 (±5)
Ever hand or wrist involvement, *n* (%)	64 (96)
Z-score of BA, mean (±SD)	−0.36 (±1.44)
Z-score of BHI, mean (±SD)	−0.85 (±1.15)^b^

^a^ANA, Antinuclear antibody; BA, Bone age; BHI, Bone Health Index; CHAQ, Child Health Assessment Questionnaire; DMARD, Disease-modifying antirheumatic drug; ESR, Erythrocyte sedimentation rate; JADAS, Juvenile Arthritis Disease Activity Score; JIA, Juvenile idiopathic arthritis; MTX, Methotrexate; RF, Rheumatoid factor; VAS, Visual analogue scale; Z-score, Standard deviation compared with healthy population. ^b^Significantly different from 0 at the *P* < 0.05 level.

Bone maturation was delayed compared with the normal population, but not significantly (mean Z-score of bone age = −0.36 (±1.44), *P* = 0.051). Bone maturation was greatly impaired (below −2 standard deviations (SD)) in eight patients, and three patients had highly accelerated bone maturation (above +2 SD). The mean Z-score of bone age was strongly influenced by one patient with a very high bone age (+5 SD), who had long-standing severe polyarticular enthesitis-related arthritis. When this patient was left out of the analysis, the mean bone maturation of the remaining patients was significantly delayed (mean Z-score of bone age = −0.45 (±1.28), *P* = 0.008). Compared with the normal population, the cBMD was lower (mean Z-score of BHI = −0.85 (±1.15), *P* < 0.001). Ten patients had a Z-score of BHI less than −2 SD.

### Regression analysis

A univariate linear regression analysis of bone age and BHI was performed to investigate which variables were associated with the Z-score of bone age and the Z-score of BHI (Tables 2 and 3). Glucocorticoid use was associated with a lower Z-score of bone age (0.79 SD, *P* = 0.028). Only male gender was significantly associated with the Z-score of BHI; being a boy lowered the Z-score of BHI of 0.65 points (*P* = 0.021).

**Table 2:** **Univariate regression coefficients of baseline variables with Z-scores of bone age** ^**a**^

**Baseline variable**	**Estimated β**	**95% CI**	***P*** **-value**
Age	0.024	−0.116 to 0.164	0.736
Male gender	−0.190	−0.921 to 0.541	0.605
Disease duration	−0.030	−0.184 to 0.1234	0.694
CHAQ	−0.021	−0.577 to 0.534	0.940
JADAS-10	0.008	−0.065 to 0.081	0.830
Ever use of systemic glucocorticoids	−0.790	−1.492 to −0.088	0.028

^a^CHAQ, Child Health Assessment Questionnaire; JADAS, Juvenile Arthritis Disease Activity Score; Z-score, Standard deviation score compared with healthy population.

**Table 3:** **Univariate regression coefficients of baseline variables with the Z-score of Bone Health Index** ^**a**^

**Baseline variable**	**Estimated β**	**95% CI**	***P*** **-value**
Age	0.067	−0.024 to 0.159	0.147
Male gender	−0.649	−1.197 to −0.102	0.021
Disease duration	0.040	−0.080 to 0.160	0.505
CHAQ	0.008	−0.420 to 0.436	0.971
JADAS-10	−0.036	−0.094 to 0.023	0.224
Ever use of systemic glucocorticoids	−0.340	−0.904 to 0.224	0.233

^a^CHAQ, Child Health Assessment Questionnaire; JADAS, Juvenile Arthritis Disease Activity Score; Z-score, Standard deviation score compared with the healthy population.

## Discussion

Application of the BoneXpert automated method for assessing bone age and cBMD in JIA patients proved to be feasible. Its use was easy and fast, and the program rejected few radiographs. The Z-scores of bone age and BHI were found to be impaired in the population of JIA patients evaluated in this study compared with a healthy population.

In addition to its validation in a healthy pediatric population [7,8], bone age measurement using BoneXpert has been evaluated in pediatric patients of short stature, children with precocious puberty and patients with congenital adrenal hyperplasia [11-13]. In these patient groups, in whom bone maturation is likely to be affected, the BoneXpert bone measurement method proved feasible. We had anticipated a higher number of rejections by the BoneXpert program relating to growth abnormalities, periarticular abnormalities and deviation of bone age commonly found in severely affected JIA patients [14-17]. Unexpectedly, BoneXpert rejected no radiographs because of these abnormalities. Besides the two rejections due to poor image quality, only one radiograph with extremely accelerated bone maturation resulted in absence of a Z-score of BHI. The low rejection rate is advantageous; however, one has to take into account that patients outside the age ranges of the program had to be excluded, who were composed mostly of older patients (older than 15 years of age for girls and older than age 17 years for boys). For follow-up of patients, it would be useful if a BHI reference existed for children who have reached skeletal maturity. Besides a low rejection rate, the method also showed a very good agreement between left- and right-hand assessment and two repeated measurements of the left-hand radiograph. The excellent agreement of the repeated measurement of one radiograph is to be expected, whereas BoneXpert is an automated computer technique. Other methods used to determine reliability, such as analysis of two radiographs of the same hand of the same patient at one time point, could not be performed, because these radiographs were not available. Bone maturation and cBMD were found to be impaired, as was expected for this group of JIA patients [1,2,17]. A regression analysis showed that delayed bone age was associated with glucocorticoid use and that lower BHI was associated with male gender. The association between delayed bone age and glucocorticoid use was not unexpected, as numerous studies have shown that glucocorticoid use may slow longitudinal bone growth and growth plate senescence [18,19]. In other earlier studies, not only bone age but also impaired BMD has been associated with the use of glucocorticoids [1,20,21]. In the present study, this was not the case; the only variable in the regression analysis significantly associated with cBMD was male gender. The lack of association between glucocorticoid use and cBMD could be due to the broad definition of glucocorticoid use (that is, cumulative dose was not taken into account) and the relatively small size of our study population. On the other hand, several randomized trials in adults with rheumatoid arthritis have shown that glucocorticoids can decelerate the loss of hand BMD [22,23]. Although rheumatoid arthritis and JIA are two different entities, a similar protective effect of glucocorticoids on hand BMD may have played a role in our population. The association with male gender is less easy to interpret. It was previously shown that differences exist between healthy boys and girls in BMD of the forearm, with boys having higher BMD of the forearm than girls [24]. This difference is not associated with body mass index, but is likely to be associated with other factors. The same group also found an association between physical activity and BMD of the forearm, combined with the finding that boys are more physically active than girls [25]. The difference in BHI between boys and girls in the current studies might therefore be explained by low physical activity due to disease activity and by boys being relatively less physically active compared to their healthy peers than girls. The link between physical activity and lower cortical thickness of the forearm was also hinted at in a study in pediatric Crohn’s disease patients. In that study, a lower cortical thickness was also found in boys, which improved with treatment, possibly because patients also increased their daily physical activities with their response to treatment [26].

The BoneXpert method is used to measure bone age and BHI. With respect to bone age, the method can be considered feasible for future use in multicenter or longitudinal follow-up studies in JIA patients, because of its easy use, high precision and small differences between left- and right-hand radiographs [8]. Besides its application in research, the BoneXpert method can also potentially be of use in clinical practice. The automatic determination of bone age and cBMD is less costly than other methods and is time-saving for both pediatric rheumatologists and radiologists. Moreover, only one hand—either right or left—needs to be analyzed, which increases the feasibility of use in daily practice (unless there is an extreme clinical discrepancy between left and right).

The other major outcome variable, the BHI, needs more validation studies before it can be used in research and clinical practice. The DXR method for the assessment of cBMD, used by BoneXpert, has been compared to DXA in several patient groups, including patients with inflammatory bowel disease [27]. In these patients, the correlation between DXR and DXA was found to be moderate to good. In JIA patients, however, cBMD of the hand may be influenced by local inflammation, possibly resulting in a lower correlation with generalized BMD, as shown in adult patients with rheumatoid arthritis [28]. If BHI can be used as a proxy for generalized BMD in JIA patients, further validation in this population is needed, including a comparison with other BMD measurement techniques. This is complicated by the fact that there are different methods used to determine BMD without consensus on the gold standard, although DXA is the most widely utilized technique.

### Limitations

This study is limited by its observational design and the very specific population derived from the ABC register. These factors introduce a selection bias, which limits generalization to the full JIA population. However, patients included in the ABC register are considered to be the most severely affected patients because of their eligibility for biologic treatment; therefore, these patients are most likely to have structural bone damage. It can be assumed that if the BoneXpert method can be applied in these patients, it can be used in all JIA patients. Most radiographs used in this study were digital; however, some conventional radiographs were included. BoneXpert works less well with these digitized images, as demonstrated by the two radiographs that were rejected by the program. Given the widespread use of digital radiology throughout Europe, this will not be a problem in future studies.

## Conclusions

To our knowledge, this study is the first in which the BoneXpert automated determination of bone age and cBMD has been evaluated in JIA patients. The method proved feasible and easy to use, even in severely affected JIA patients, provided that radiographs were of reasonable quality and patients were within the age ranges of the BoneXpert program. This method can be implemented in clinical practice for the determination of bone age in JIA patients. It needs further validation for the determination of bone health, including comparison with existing methods for the determination of BMD.

## Additional files

Additional file 1:
**Flowchart of patient selection.**
Additional file 2:
**Bland-Altman plots of the agreement between left and right hands.**

## Abbreviations

BA: Bone age
BHI: Bone Health Index
(c)BMD: (Cortical) bone mineral density
CHAQ: Child Health Assessment Questionnaire
DMARD: Disease-modifying antirheumatic drug
DXA: Dual-energy X-ray absorptiometry
DXR: Digital X-ray radiogrammetry
ESR: Erythrocyte sedimentation rate
ICC: Intraclass correlation coefficient
JADAS: Juvenile Arthritis Disease Activity Score
JIA: Juvenile idiopathic arthritis
MTX: Methotrexate
VAS: Visual analogue scale
