# Supplementary material for: Automated determination of bone age and bone mineral density in patients with juvenile idiopathic arthritis: a feasibility study
Source: Arthritis Res Ther. 2014 Aug 27;16(4):424. doi: 10.1186/s13075-014-0424-1 (PMC4293113; doi:10.1186/s13075-014-0424-1)
Supplement: Additional file 1: — Flowchart of patient selection. [file 13075_2014_424_MOESM1_ESM.doc]

**ADDITIONAL FILE 1**

Flowchart of the patient selection


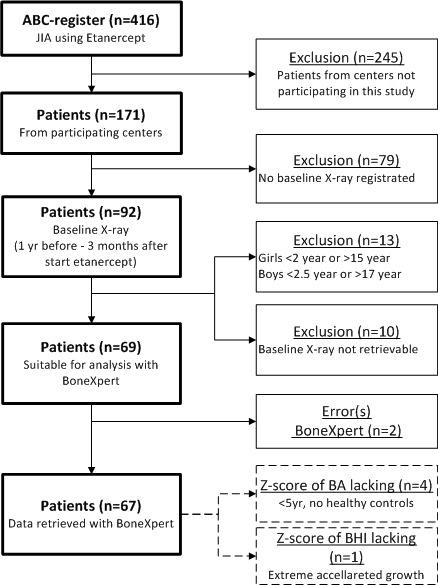


ABC-register = Arthritis and Biologicals in Children register; JIA = juvenile idiopathic arthritis; BA = bone age; BHI = Bone Health Index.
