# Supplementary material for: Automated determination of bone age and bone mineral density in patients with juvenile idiopathic arthritis: a feasibility study
Source: Arthritis Res Ther. 2014 Aug 27;16(4):424. doi: 10.1186/s13075-014-0424-1 (PMC4293113; doi:10.1186/s13075-014-0424-1)
Supplement: Additional file 2: — Bland-Altman plots of the agreement between left and right hands. [file 13075_2014_424_MOESM2_ESM.doc]

**ADDITIONAL FILE 2 -** Bland-Altman plots depicting the agreement between the left and right hand radiograph


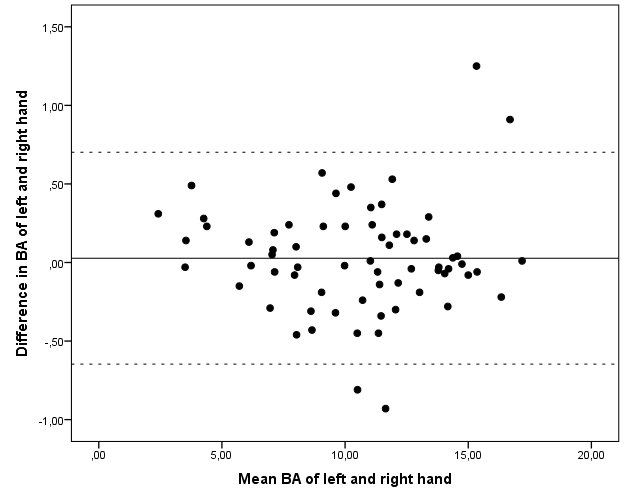


**Figure 1.** Bland-Altman plot depicting the agreement of the bone age (BA) between the left and right hand radiograph. The horizontal line is the mean of the repeated measurements, the dotted line is +2SD.


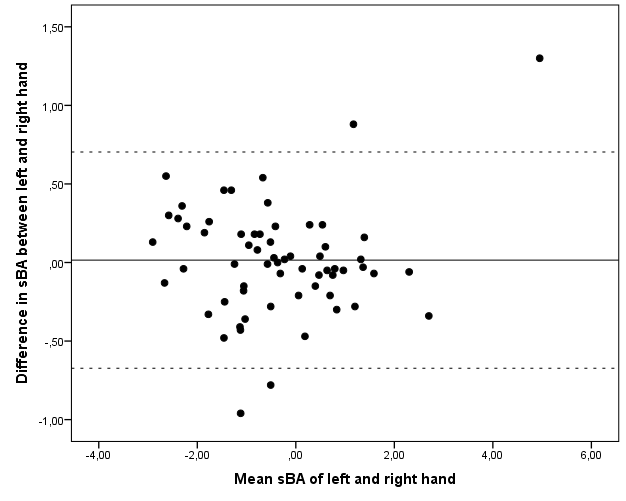


**Figure 2.** Bland-Altman plot depicting the agreement of the z-score of the bone age (BA) between the left and right hand radiograph. The horizontal line is the mean of the repeated measurements, the dotted line is +2SD.


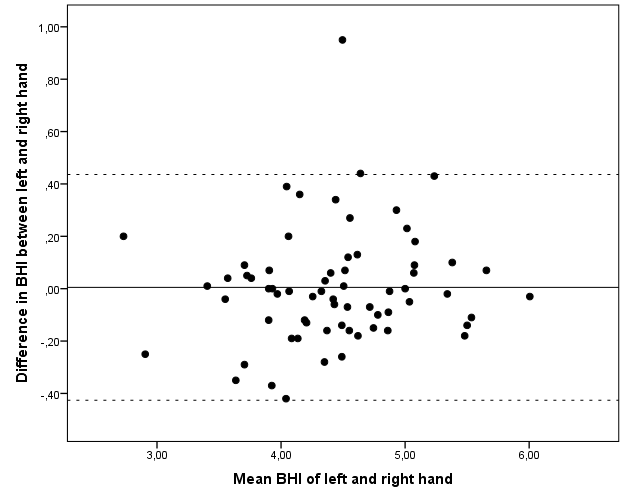


**Figure 3.** Bland-Altman plot depicting the agreement of the Bone Health Index (BHI) between the left and right hand radiograph. The horizontal line is the mean of the repeated measurements, the dotted line is +2SD.


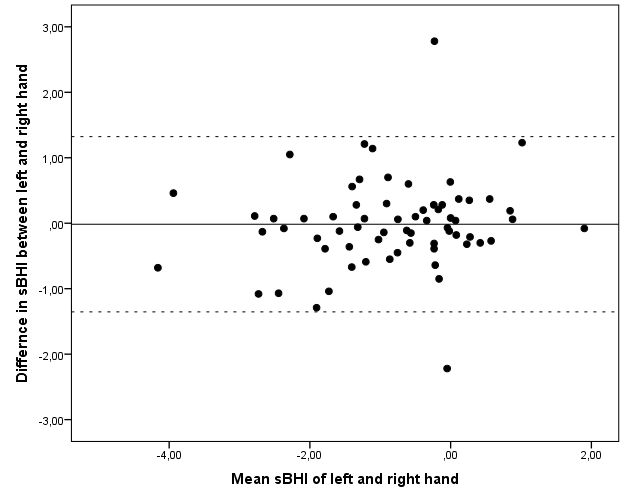


**Figure 4.** Bland-Altman plot depicting the agreement of the z-score of the Bone Health Index (BHI) between the left and right hand radiograph. The horizontal line is the mean of the repeated measurements, the dotted line is +2SD.
